# Integrating Artificial Intelligence Tools in the Clinical Research Setting: The Ovarian Cancer Use Case

**DOI:** 10.3390/diagnostics13172813

**Published:** 2023-08-30

**Authors:** Lorena Escudero Sanchez, Thomas Buddenkotte, Mohammad Al Sa’d, Cathal McCague, James Darcy, Leonardo Rundo, Alex Samoshkin, Martin J. Graves, Victoria Hollamby, Paul Browne, Mireia Crispin-Ortuzar, Ramona Woitek, Evis Sala, Carola-Bibiane Schönlieb, Simon J. Doran, Ozan Öktem

**Affiliations:** 1 Department of Radiology, University of Cambridge, Cambridge CB2 0QQ, UK; 2 Cancer Research UK Cambridge Centre, Li Ka Shing Centre, Cambridge CB2 0RE, UK; 3 National Cancer Imaging Translational Accelerator (NCITA) Consortium, UK; 4 Department of Applied Mathematics and Theoretical Physics, University of Cambridge, Wilberforce Road, Cambridge CB3 0WA, UK; 5 Department for Diagnostic and Interventional Radiology and Nuclear Medicine, University Hospital Hamburg-Eppendorf, 20246 Hamburg, Germany; 6 Jung Diagnostics GmbH, 22335 Hamburg, Germany; 7 Cancer Imaging Centre, Department of Surgery & Cancer, Imperial College, London SW7 2AZ, UK; 8 Cambridge University Hospitals NHS Foundation Trust, Cambridge CB2 0QQ, UK; 9 Division of Radiotherapy and Imaging, Institute of Cancer Research, London SW7 3RP, UK; 10 Department of Information and Electrical Engineering and Applied Mathematics (DIEM), University of Salerno, 84084 Fisciano, Italy; 11 Office for Translational Research, School of Clinical Medicine, University of Cambridge, Cambridge CB2 0SP, UK; 12 Research and Information Governance, School of Clinical Medicine, University of Cambridge, Cambridge CB2 0SP, UK; 13 High Performance Computing Department, University of Cambridge, Cambridge CB3 0RB, UK; 14 Department of Oncology, University of Cambridge, Cambridge CB2 0XZ, UK; 15 Research Centre for Medical Image Analysis and Artificial Intelligence (MIAAI), Department of Medicine, Faculty of Medicine and Dentistry, Danube Private University, 3500 Krems, Austria; 16 Dipartimento di Scienze Radiologiche ed Ematologiche, Universita Cattolica del Sacro Cuore, 00168 Rome, Italy; 17 Dipartimento Diagnostica per Immagini, Radioterapia Oncologica ed Ematologia, Policlinico Universitario A. Gemelli IRCCS, 00168 Rome, Italy; 18 Department of Mathematics, KTH-Royal Institute of Technology, SE-100 44 Stockholm, Sweden

**Keywords:** artificial intelligence, cancer research, imaging, clinical integration, radiomics

## Abstract

Artificial intelligence (AI) methods applied to healthcare problems have shown enormous potential to alleviate the burden of health services worldwide and to improve the accuracy and reproducibility of predictions. In particular, developments in computer vision are creating a paradigm shift in the analysis of radiological images, where AI tools are already capable of automatically detecting and precisely delineating tumours. However, such tools are generally developed in technical departments that continue to be siloed from where the real benefit would be achieved with their usage. Significant effort still needs to be made to make these advancements available, first in academic clinical research and ultimately in the clinical setting. In this paper, we demonstrate a prototype pipeline based entirely on open-source software and free of cost to bridge this gap, simplifying the integration of tools and models developed within the AI community into the clinical research setting, ensuring an accessible platform with visualisation applications that allow end-users such as radiologists to view and interact with the outcome of these AI tools.

## 1. Introduction

Medical imaging is routinely used in clinical centres to detect and monitor various diseases. In particular, it is one of the pillars for the diagnosis and treatment monitoring of cancer, with computed tomography (CT) being one of the standard-of-care imaging methodologies.

The field of medical imaging has undergone a rapid evolution following the development of new instrumentation over the last several decades. But although imaging is one of the core components of patient care, and despite the multitude of related advancements in mathematical and computation methods for image analysis, in practice, the analysis of radiological images in the clinical setting is still often limited to a manual and semi-quantitative assessment.

Quantitative analysis of radiological images can support decision making and improve patient care. One example of this is the radiomics approach, which extracts features from image pixel data, conveying information not identifiable by eye about their distribution of grey levels, intensity or heterogeneity [1,2,3]. Multiple studies have focused on assessing the clinical relevance of such features and using them in the training of machine learning (ML) models for disease classification or prediction of tumour evolution or treatment response, with promising results [4].

Computational methods play a key role in both *image reconstruction* and *image analysis*. Image reconstruction refers to theory and algorithms for computationally recovering the (medical) image from measured sensor data. For example, a magnetic resonance imaging (MRI) reconstruction will turn data collected from a radiofrequency head coil of an MR scanner into the image of a head that can be readily interpreted by a human observer. Image reconstruction often takes place on the scanner itself, but offline reconstruction can be very important for specialist research applications. Image analysis refers to computational methods for the downstream processing of images that aims to extract clinically relevant information from them. The last decade has seen an increased effort in developing artificial intelligence (AI)-based methods for image analysis, and in particular methods that build on deep neural networks (DNNs). These DNN-based models can, in specific settings, exhibit excellent performance that rivals human performance [5].

The potential of the aforementioned research on AI models for image reconstruction and image analysis is best realised if these are developed, tested and deployed in close collaboration with health care professionals in a clinical setting [6]. However, most developments on mathematical and computational methods for medical images are still far from being deployed in the clinical setting. Integrating AI-based support systems for cancer detection and treatment into the clinical setting, creating an automated end-to-end pipeline for the optimisation of image reconstruction and the extraction of clinically relevant information, is the ultimate goal of our interdisciplinary team. Here, we present the first stage: integrating deep learning (DL)-based automated tumour segmentation and ML-based predictive models into the clinical research setting, using high grade serous ovarian cancer (HGSOC) as a specific use case for which no tools are openly available yet. HGSOC is the leading cause of mortality associated with gynaecologic malignancies; the five-year survival rate for those with metastatic disease is under 30% [7].

Our aim is to establish a workflow for integrating AI-based inference models (which can be for reconstruction, segmentation, prediction, etc.) into the clinical research setting, to make such algorithms and models available to radiologists as end-users and to aid AI developers to test, validate and improve them with real clinical data. In this paper, we will describe the general elements of the pipeline first and then focus on the concrete use case of HGSOC as an example, using the in-house methods we have developed, namely a DL-based model for automated segmentation followed by an ML-based radiomics prediction of chemotherapy treatment response.

## 2. Materials and Methods

This section outlines how to set up a technical platform for creating and deploying AI-based workflows for image-guided clinical research, which caters to *both* end-users (clinicians, in particular radiologists) and developers of AI models (mathematicians, computer scientists and data analysts), with CT in oncology serving as the exemplary use case. Building such a platform is a huge task that requires vast software engineering resources, and the strategy is therefore to build on existing initiatives for platforms that follow a similar design philosophy. The desired properties required are:

- The seamless integration of AI models that allows for their agile development and upgrading.
- Being based on open-source software to (i) enable academic reproducibility of clinical AI research, (ii) reduce the cost of research and (iii) ensure that continued research in the area is not contingent on collaborations with specific commercial partners or vendors.
- Zero footprint, to avoid version mismatches and incompatibilities, and the need to install software locally.
- Being built on a user-friendly and intuitive application that allows the end-user to view and interact with the radiological images.

### 2.1. AI Integration for Developers

A key functionality aimed at developers is to offer scalable application programming interfaces (APIs) for integrating bespoke AI models into existing workflows. Some openly available platforms are the https://www.nvidia.com/en-gb/clara/ (accessed on 23 June 2023) Clara Medical Imaging Platform by NVIDIA, https://github.com/microsoft/InnerEye-DeepLearning (accessed on 23 June 2023) InnerEye-DeepLearning by Microsoft or GaNDLF [8]. We used the Clara Medical Imaging Platform, which is highly flexible and includes an open-source image-processing framework suited for AI models in medical imaging (https://docs.nvidia.com/monai/ (accessed on 23 June 2023) Medical Open Network for AI (MONAI)) along with software development kits (SDKs) for training (Clara Train SDK) and deployment (Clara Deploy SDK).

In addition, thanks to its integration with XNAT and the user interface provided by the OHIF viewer plugin, it also offers means to make such algorithms and models available to radiologists as end-users, as we will describe.

#### 2.1.1. Imaging Database and Visualisation

The first element necessary for such a pipeline is a safe and robust framework to store the necessary clinical files in an accessible manner. For images, these are files in the digital imaging and communications in medicine (DICOM) http://medical.nema.org/ (accessed on 23 June 2023) format, but the framework should also be able to include other formats. We chose the Extensible Neuroimaging Archive Toolkit (XNAT) [9] as our framework. It is a widely used open-source framework that provides a web-based interface to an underlying database that is especially suitable for imaging research in clinical multi-centre projects. XNAT provides a user interface that allows users to access data simply via a web browser, an Internet connection and personal login details. The desired platform built on XNAT ensures the import, archiving, processing and secure data distribution required for the study of clinical data, the aim of our pipeline. Detailed instructions to install and set up an XNAT server are available in the https://wiki.xnat.org/documentation/xnat-administration (accessed on 23 June 2023) official XNAT documentation, which was followed for the work presented here.

One of the most popular XNAT plugins integrates the Open Health Imaging Foundation (OHIF) viewer [10], creating a DICOM visualisation environment directly within the web browser used by the end-user. This requires only the plugin to be installed in the repository server where XNAT is deployed, giving access to the viewer to all users without them having to install any software (zero footprint). The installation of the OHIF viewer plugin to XNAT is extremely easy, as it only requires copying the plugin .jar file in the appropriate folder (e.g., /data/xnat/home/plugins) and restarting the *tomcat* service. More details can be found in the https://wiki.xnat.org/documentation/xnat-ohif-viewer (accessed on 23 June 2023) official documentation followed.

#### 2.1.2. AI Inference and Training

There are different ways in which we can interact with our pipeline, which vary in terms of the level of user interface availability. On one extreme, we have the possibility to incorporate and run AI models directly from the visualisation framework created by the OHIF viewer plugin, which allows end-users to interact with the input and output of the model inference without any knowledge of the specifics of the code running in the background. This will be demonstrated in the final part of this paper.

**Figure 1:**
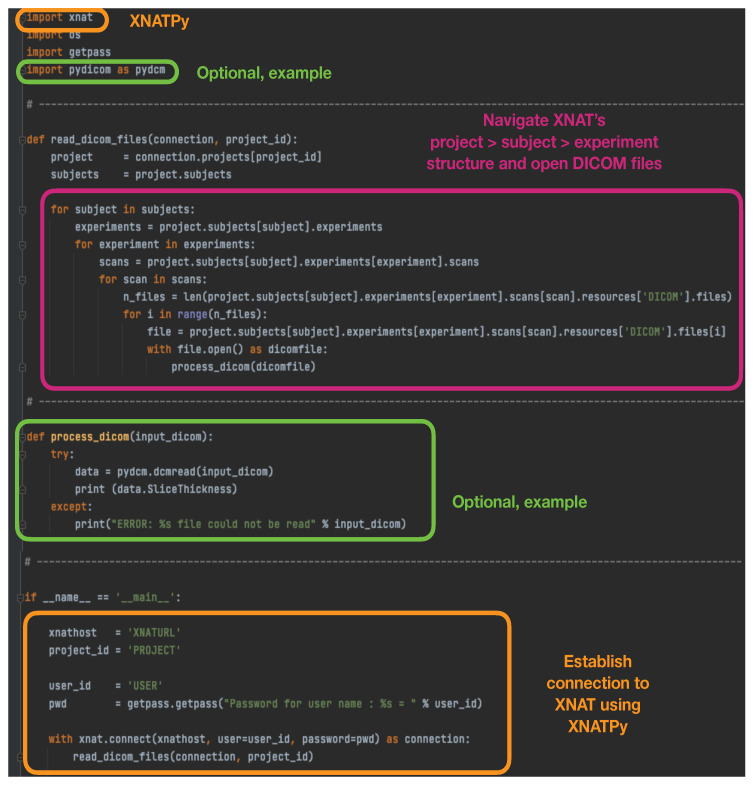
Example script using XNATPy to establish a connection with the XNAT database, open stored DICOM files and perform an action on them.

On the other extreme, the https://xnat.readthedocs.io (accessed on 23 June 2023) XNATPy Python interface provides a client that exposes XNAT objects and functions as Python objects and functions. Through this method, it is very easy to establish with only a few lines a connection to XNAT to read existing files in the database, and to create and upload new files, imaging sessions, subjects, etc. An example script that simply establishes a connection with the XNAT database, opens stored DICOM files and performs an action on them can be found in Figure 1.

Between these two levels of interface, tools can also be developed and deployed with (i) with the https://developer.nvidia.com/industries/healthcare (accessed on 23 June 2023) NVIDIA Clara Application Framework and (ii) XNAT’s capability of running containerised software. For the latter, XNAT has a dedicated plugin for controlling and running containers using Docker [11] and Docker Swarm. Instructions to build, set up and launch containers within the XNAT platform can be found in the https://wiki.xnat.org/container-service/ (accessed on 23 June 2023) XNAT container service documentation, with illustrative examples showing how containers can be run from specific projects and imaging sessions within the database. This method provides APIs to easily interact with the container service, managing containers, configurations and commands.

In general, XNAT provides an ideal environment for image processing, but it is important to highlight that it is not limited to DICOM files, as it provides a structured storage system for any type of files, which can in turn be used as the input to AI models, and also stored back to the database with the output of the model. In particular, we stored raw files containing sinograms extracted from the CT scanner together with the reconstructed images, which could themselves be used for AI-based image reconstruction, using different containerised reconstruction models.

#### 2.1.3. Automated Segmentation Tools

In order to integrate automated segmentation methods, we selected tools developed by NVIDIA that are publicly available and provide client APIs compatible with a range of viewers, including Slicer [12] and OHIF, that facilitate the interaction with the platform for AI developers and the integration and testing of models. Originally, our pipeline was developed using the AI-assisted annotation (AIAA) SDK described in the https://docs.nvidia.com/clara/clara-train-sdk/aiaa/index.html (accessed on 23 June 2023) AIAA documentation, which is part of the Clara framework.

AIAA runs easily as a Docker container, and once the AIAA server is deployed and connected to XNAT, models are automatically available for end-users in general or on a project-level basis, as selected by the XNAT administrator. Crucially, this AIAA framework allows XNAT administrators to easily add new models, which is a key advantage of our pipeline. The mask containing the semantic segmentation created by the DL-based model inference is seamlessly returned to the viewer and immediately overlaid on the input radiological images. Control then passes back to the OHIF viewer, which allows end-users to interact with the segmentations, scrolling, editing or deleting them as appropriate, and saving them directly into XNAT, where they become searchable data objects. Detailed instructions on how to install the AIAA server version used in this paper, and how to upload models, have been added to the https://wiki.xnat.org/documentation/xnat-ohif-viewer/xnat-ohif-plugin-installation-and-administration-124813344.html (accessed on 23 June 2023) XNAT’s online documentation library.

Subsequent work by our team has added compatibility of the inference functionality of the https://github.com/Project-MONAI/MONAILabel (accessed on 23 June 2023) MONAILabel project to the XNAT-OHIF plugin. This follows upgrades by NVIDIA on their AIAA tools, replacing the original, closed-source commercial library with the PyTorch-based open-source framework for healthcare imaging MONAI [13]. This holds out the potential of training and re-training models in response to real-time inputs by end-users directly through their interface with OHIF viewer. However, this aspect of the workflow remains as a work in progress.

New models can be incorporated to the pipeline through the AIAA server API web-page, or with the command line via a https://curl.se/ (accessed on 23 June 2023) *curl* command. In our case, we incorporated our pre-trained models using the latter, for which the following elements are necessary:

**(i) A self-contained library of the new model.** It must include pre-trained weights and any additional parameters needed at runtime. Depending on the AIAA version used, a *.ts* (PyTorch TorchScript) file containing the model library might be necessary during the upload to AIAA. However, once the library is installed and available for the Docker container in the library path, changes can be carried out directly in the installed library. For those models that cannot be exported as *.ts* due to their characteristics, we tested uploading a simplified or even dummy *.ts* file with curl, which informs AIAA that a new model exists.

**Figure 2:**
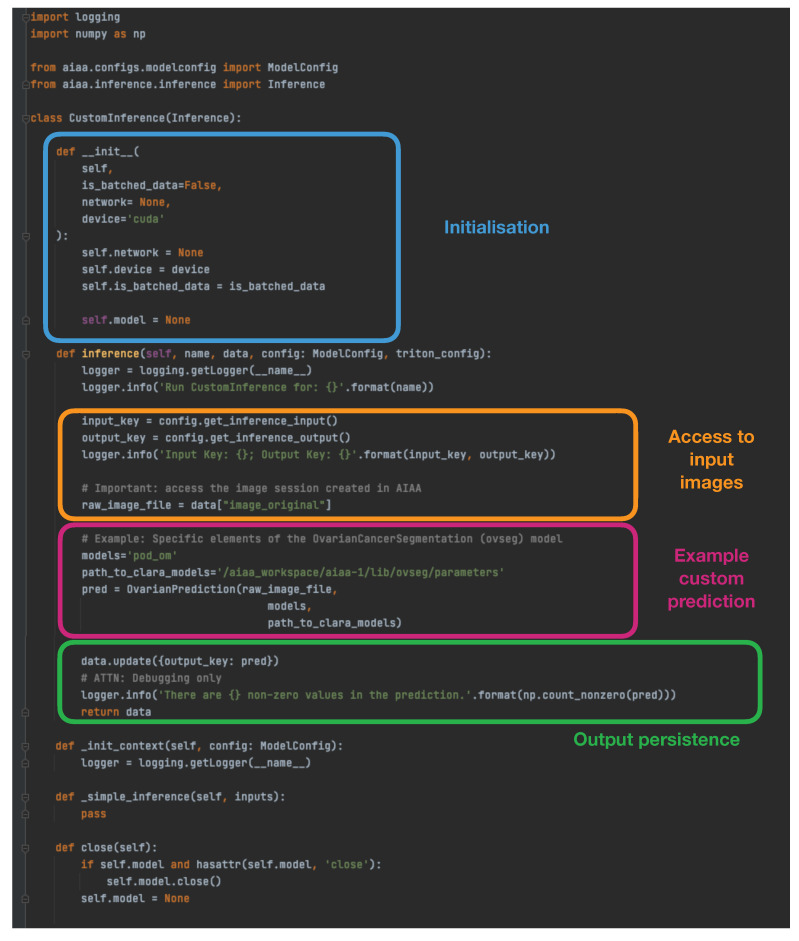
Example custom inference implemented for the ovarian cancer segmentation to integrate in the AIAA framework, showing the I/O logic necessary before and after running the algorithm.

**(ii) An appropriate custom inference to communicate the model I/O within the overall context of Clara/AIAA.** Based on the simple examples provided by NVIDIA in https://docs.nvidia.com/clara/clara-train-sdk/aiaa/byom/byoi.html (accessed on 23 June 2023) their documentation, we built the CustomInference needed for our ovarian cancer example, simply stating the I/O and performing all the logic inside a wrapper called *OvarianPrediction* defined within the custom library, as shown in Figure 2.

**(iii) A** ***.json*****configuration file.** Importantly, it should contain:

- Type: *Segmentation*, *Annotation* or *Deep Grow* (see 2.2 D).
- Labels: the output that will be retrieved from the model, in the expected order and with the names to be displayed.
- Pre-transforms: must include at least the loading of images (via MONAI).
- Inference: including I/O (like Figure 2).
- Post-transforms: must include at least a transformation to NumPy array (via MONAI) needed for the final writing of the output.
- Writer: using the predefined AIAA method.

**Figure 3:**
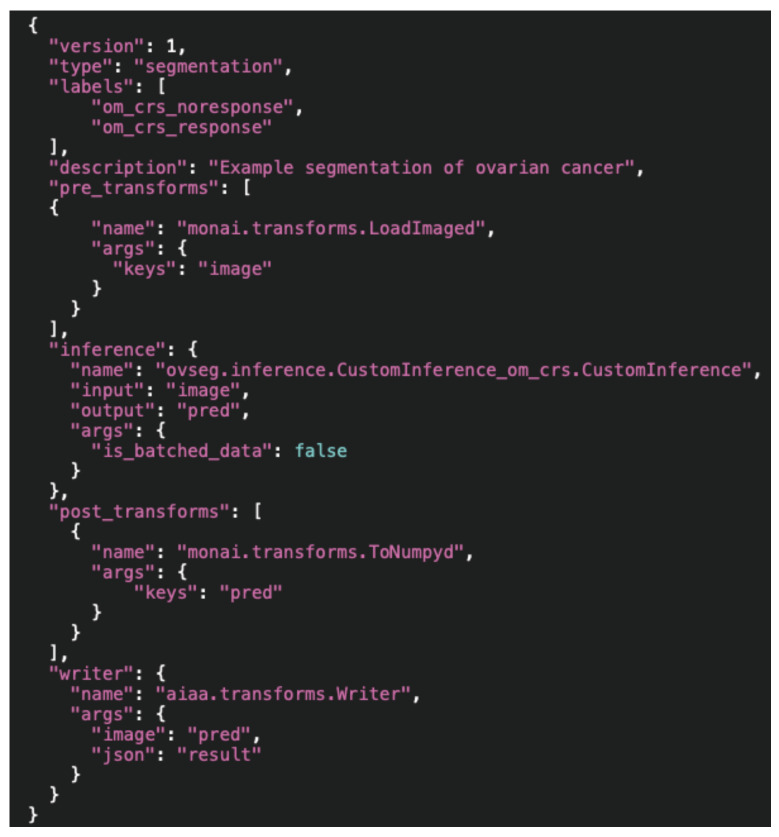
Simple configuration file showing the I/O elements necessary for a custom model with two possible outcomes (treatment response and non-response) to interface with AIAA/MONAI, keeping all the logic in the custom inference (CustomInference_om_crs.CustomInference).

Hence, the simplest configuration file for our example ovarian cancer segmentation that considers two possible outcomes (treatment response and non-response), and in which all the logic (including specific pre- and postprocessing steps) happens within the inference of our custom model (therefore with just the exception of the I/O elements necessary to interface with AIAA/MONAI) would look like the one in Figure 3.

Once these elements have been created, an example of the *curl* command to upload or incorporate the new model into the AIAA server would look like: 

curl -X PUT "http://$AIAA_SERVER_IP/admin/model/$model_name?native=true" \\
-F "config=@$model_path/model_config.json;type=application/json" \\
-F "data=@$model_path/model.ts”

### 2.2. AI Integration for End-Users

Usability is key to ensuring that end-users (e.g., radiologists and clinicians) can interact with the AI models in an effective and simple way, without needing programming skills. The XNAT ecosystem offers such an interaction with tools and user interfaces that enable running the AI models both interactively and automatically. Users can interact with the output of the models, and ultimately take active part in the development of such and new models at their own institutions, using their own patient data with a focus on their own clinical questions. This section outlines a high-level overview of the main features provided by the our platform in terms of AI integration and usability for end-users.

#### 2.2.1. Imaging Database and Visualisation

**Figure 4:**
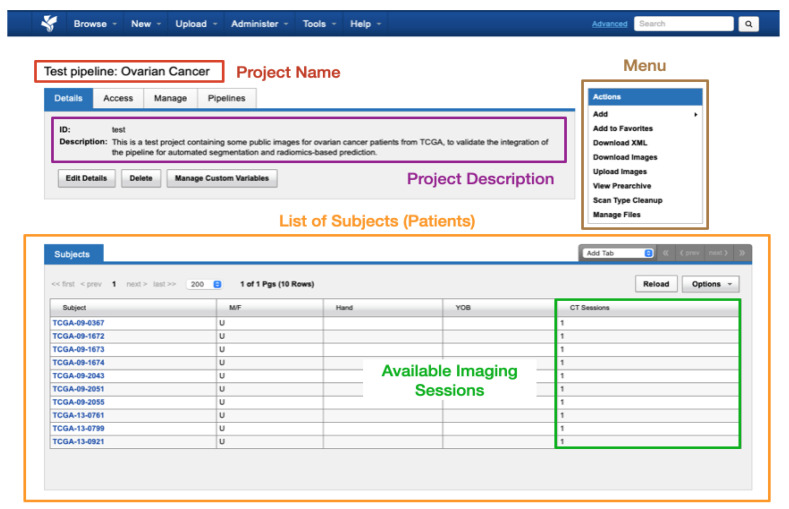
Schematic view of the display of a test project in XNAT.

Data within XNAT is organised into projects where each research study can be assigned its own project. Access to a project’s data can be controlled by the administrator assigning access permissions to authorised users. Within each project, the data are divided into subjects (typically patients) that in turn are composed of one or multiple experiments (equivalent to imaging sessions of a given modality, with all common DICOM modalities supported such as CT, MRI, radiography, etc.). A schematic view of the display of a test dummy project in XNAT can be found in Figure 4.

For end-users, the XNAT-OHIF Viewer plugin provides, besides the standard features for medical image viewing (such as windowing, scroll, multi-viewports, and a cine player), other advanced features such as multi-modal image fusion and multi-planar reformatting. It also supports different annotation tools, including mask segmentation, contour delineation and measurements.

#### 2.2.2. AI Inference and Training

The viewer allows end-users to run AI models, as it will be shown while viewing images, as well as visualise the segmentation output overlaid on images and edit the segmentation interactively to adjust or fine-tune the output. The end-user can then export the output in a DICOM-compliant format to be stored directly on XNAT, as well as import the segmentation data of a previously stored run result. The inference can also be run in batches using containerised software.

#### 2.2.3. Automated Segmentation Tools

The platform created provides a pre-trained NVIDIA organ and image modality-specific models available in their catalogue, for example, for CT images of the spleen, the liver, or the pulmonary findings of COVID-19. Such models are able to return multiple labels (e.g., different organs individually or a given organ + tumour) and they can be classified in three different categories:

- *Segmentation* models, to produce segmentations in a fully automated manner, without any user input.
- *Annotation* models, semi-automated models requiring user input (selected seed points defining a bounding box).
- *Deep Grow* models, interactive models that follow the “clicks” by the user to define and iteratively refine the region of interest (foreground vs. background).

**Figure 5:**
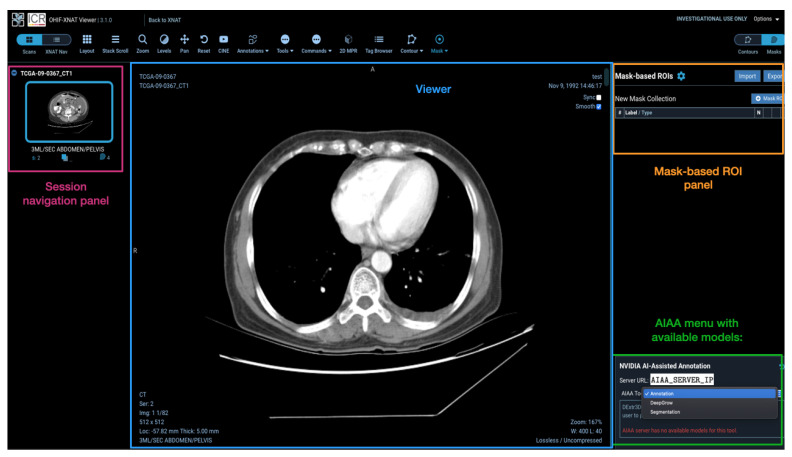
View of a CT imaging session in the OHIF viewer plugin to XNAT with the mask menu on the right-hand side presenting the connection to the AIAA server established and the drop-down menu with the categories of available AI models.

Models installed on the AIAA server by system administrators to the AIAA server are available to end-users and displayed in a drop-down menu in the OHIF viewer plugin when an imaging session is opened, as illustrated in Figure 5, divided in each of those three categories.

### 2.3. Example Use Case: Ovarian Cancer Segmentation and Response Prediction

**Figure 6:**
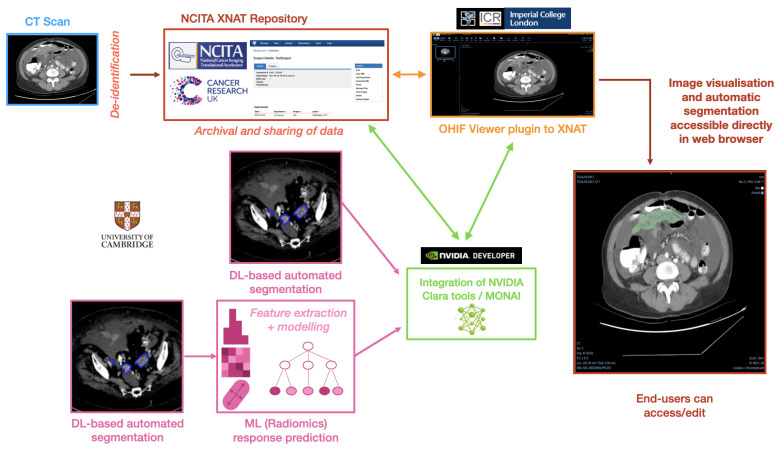
Overview of the pipeline created for the ovarian cancer segmentation and response prediction use case, showing its multiple components that seamlessly integrate AI models into visualisation tools.

As a concrete example, this section describes the pipeline framework in the context of ovarian cancer, integrating AI methods for automated tumour segmentation and prediction of chemotherapy treatment response. In order to achieve the desired pipeline framework, we integrated different elements represented in the schematic overview of the pipeline that can be found in Figure 6, highlighting its multiple components.

High-grade serous ovarian cancer is typically diagnosed at a late stage, in which the disease has spread across the whole abdomen and beyond. To distinguish between lesions found in different locations, we introduced different disease classes for each location and trained multiple models for the segmentation of those. A total of three models were trained targeting different lesion locations, namely (i) pelvic/ovarian and omental lesions (as suggested in [14]); (ii) abdominal lesions, namely lesions in the omentum, right- and left upper quadrant, the mesentery and the left and right paracolic gutter; and (iii) lymph nodes, namely infrarenal, suprarenal, supradiaphragmatic and inguinal lymph nodes. For each one of these three models, a *.json* configuration file and a *curl* command, as explained in the previous section, was created and used to upload each model independently, specifying the different labels of the output mask(s). This, in practice, was very simple, as the same library is shared by all of them, and the only additional code was in terms of a new *CustomInference* for each one with the appropriate settings.

The DL-based model used was a modified version of the model suggested in [14]. The model was created by extensive hyper-parameter tuning using a parametrisation suggested by the nnU-Net framework [15] as a baseline to ensure state-of-the-art performance. The architecture was chosen to be a four-stage U-Net with a ResNet [16] encoder of 1, 2, 6, and 3 blocks and 32, 64, 128, and 256 filters per stage. Before training, all volumes were first re-sized to an in-plane pixel spacing of 0.8 mm and 5 mm slice distance followed by windowing and normalisation, as suggested by nnU-Net. The training was performed using a batch size of 4, forcing one sample in each batch to be centred at a randomly chosen foreground voxel. A linear ascent plus cosine decay was chosen as a learning rate schedule with maximum 0.02. Standard stochastic gradient descent with a Nesterov’s momentum [17] of 0.98 and weight decay of $10^{-4}$ was used as the optimiser. The data augmentation applied during training was left unchanged as suggested by nnU-Net. During inference, we prevented subsampling artifacts for scans with low slice thickness, by re-sampling the volumes to 5/k instead of 5 mm slice distance. The obtained volume was split in k subvolumes by picking every *k*th slice, each of which was evaluated individually. Next, the sliding window algorithm was applied on each subvolume to predict the segmentation on the full subvolumes rather than patches. After this, the predictions of the subvolumes were merged again to one volume with slices in the corresponding positions of their image inputs. In contrast to [15], we did not apply extensive flipping as test time augmentations to reduce the computational cost. Finally, an ensemble of three identical models trained at random seeds were used as an ensemble at inference time by computing the average over the softmax outputs of the network. The conversion to integer-valued labels was performed by applying the channel-wise argmax-function. The implementation was performed using PyTorch 1.9 and is available at LINK BLINDED. It is recommended to perform the inference using a GPU compatible with CUDA 10 and at least 6 GB of VRAM.

For the present pipeline and as a proof of concept, we created an additional segmentation model using the network described previously, which outputs the segmentation of specifically the omental lesions to afterwards run an ML-based prediction of treatment response on the segmented area. Once the segmentation inference finishes and the output mask is obtained, this prediction is used to extract radiomic features using the https://pyradiomics.readthedocs.io/en/latest/ (accessed on 23 June 2023) PyRadiomics python library. The aim is to use these radiomics measurements to predict the response of the patient to neoadjuvant chemotherapy treatment (NACT) according to chemotherapy response score (CRS). CRS is the most validated early surrogate biomarker of response in HGSOC [18]; however, its primary drawback is that it can only be calculated on an omental tissue specimen after surgery. A previously validated radiomics-based method has shown to predict CRS from pre- and post-NACT CT scans without the need of surgical specimens [4]. For simplicity, the predictive model described in [4] was recreated with the selected clinically interpretable features only, using a random forest model trained and validated on the same datasets as the original work. The ML model was built and saved as a Python scikit-learn *joblib* file and loaded at runtime as a postprocessing step after the inference is obtained. The final outcome of the DL+ML model is therefore the segmentation of the omental lesions labelled with either ‘TRUE’ (green coloured segmentations) or ‘FALSE’ (red coloured segmentations) if the prediction for the patient is to respond or not, respectively, to NACT, according to the CRS.

### 2.4. Hardware and Information Governance Requirements

The different elements in Figure 6 are incorporated into the pipeline such that the several processes run unperceived by the end-user. In general terms, it requires two servers to be deployed and connected to each other:

- *Repository server*, either Linux-based or able to run a virtual machine (VM) via Vagrant or similar to deploy the XNAT-based database.
- *AI server*, with GPU access and Docker installed, to run the AIAA Docker container and the inference of the models. It also needs to have a static IP address accessible from the *repository server*.

Both servers can be hosted in the same physical or virtual machine. The platform has been tested with different configurations, with either a local, physical machine vs. a cloud-based solution for either or both the *repository* and the *AI server*, including testing with local services as well as with commercial solutions such as Amazon Web Services (AWS).

It is a requirement for our repositories to store only de-identified images; however, given appropriate security measurements, the pipeline could be replicated in an identifiable context. According to the chosen configuration, there are different possibilities as well to be in accordance with information governance requirements. The ovarian cancer use case presented here is in fact following the most challenging configuration in this respect, since it works with a cloud-based *repository server* and an independent, physical *AI server*. In this setting, and in order to prevent the transfer of DICOM images, even though de-identified at the origin, we opted to use the option of the AIAA integration in the OHIF viewer plugin that works with an intermediate file format: the DICOM images open in the viewer are buffered and a NIfTI [19] file, with only reduced metadata, is created and transferred to the *AI server* instead.

## 3. Results

The example of the ovarian cancer described in Section 2.3, including segmentation and response prediction, will be used in this section to illustrate the steps and tools created for the pipeline, applied to a specific use case.

### 3.1. Pipeline and APIs

**Figure 7:**
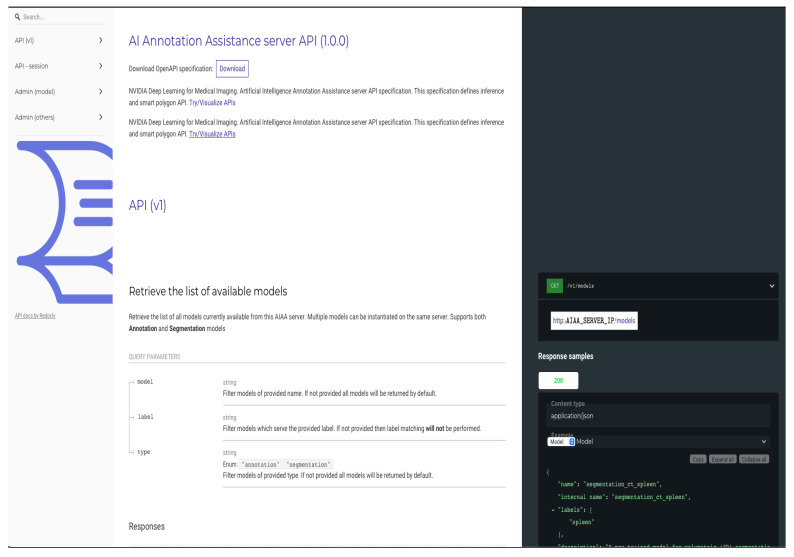
Upper portion of API web-page created by the NVIDIA AIAA Docker container and accessed in the URL of the static (public) IP address of the server.

Once the AIAA Docker container is running in the *AI server*, an API web-page is ready and can be accessed in the URL of the static (public) IP address of the server, as shown in Figure 7, accessed as http://AIAA_SERVER_IP.

**Figure 8:**
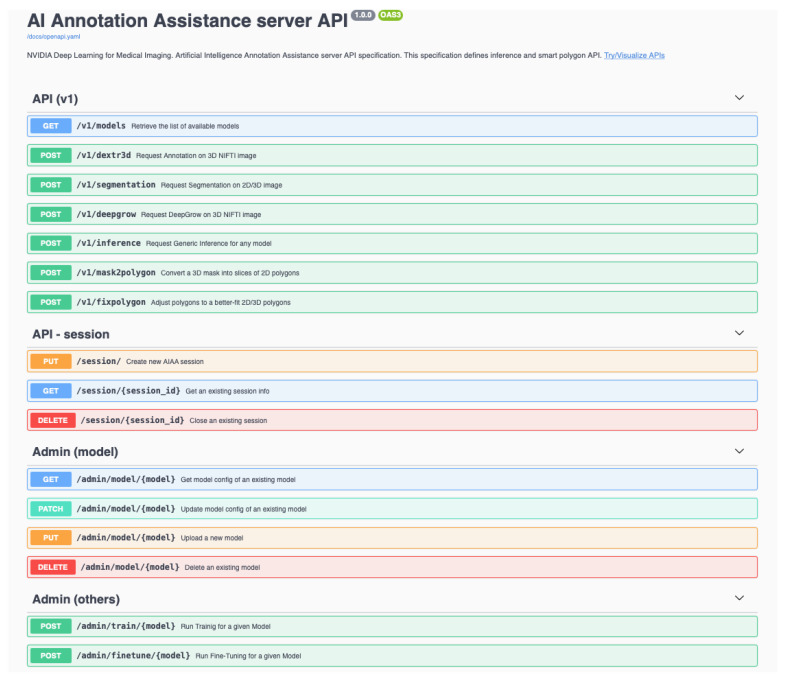
Basic administration commands (e.g., list, access, upload or delete models) accessible through the API interface.

This API interface gives access to basic administration commands (e.g., list, access, upload or delete models) that can be run interactively through this web page, as illustrated in Figure 8, accessed as http://AIAA_SERVER_IP/docs.

Any additional packages can be installed inside the AIAA Docker container by initiating a remote shell via the SSH protocol into it, provided that compatible versions of the desired libraries exist. In our ovarian cancer use case, it was necessary, for example, to install two additional Python libraries: rt-utils and PyRadiomics. When the AIAA Docker container is started, it is advisable to pass as input and mount a directory accessible from outside the container. This is where sessions (equivalent to imaging sessions) will be temporarily stored and where the libraries of our custom models will be installed, typically in a path of the form: aiaa_workspace/aiaa-1/lib/). Files can be changed or added directly in this directory from outside the container, for example, if we want to replace weights with an updated version. This can be carried out very easily, simply by adding/replacing those files, without needing to *upload* the model to AIAA again: the only necessary step for the AIAA server to pick up the changes is to restart (stop and recreate) the Docker container.

From the API web-page, it is also possible to access the logs generated by the AIAA server, containing useful information, for example, to debug if an error happens when uploading or running a model, or to print out debugging information for further developments. It can be accessed as http://AIAA_SERVER_IP/logs.

**Figure 9:**
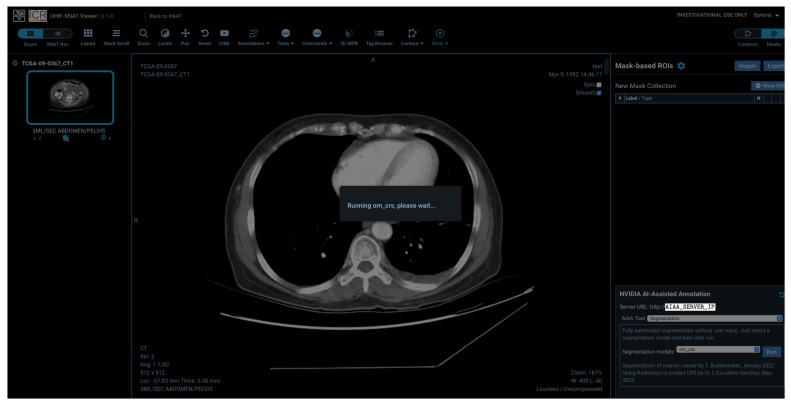
Waiting screen seen by the end-user while the session is created at the *AI server* and the AI inference process is running.

During execution, the end-user will just see a waiting screen, transparent to any processes running in the *AI server* in the meantime (e.g., segmentation model inference), as shown in Figure 9. The waiting time can take from seconds to several minutes, depending on the size of the imaging session, the Internet network, the *AI server* capacity and the complexity of the model inference running.

### 3.2. Integration of a New Model into the Pipeline

**Figure 10:**
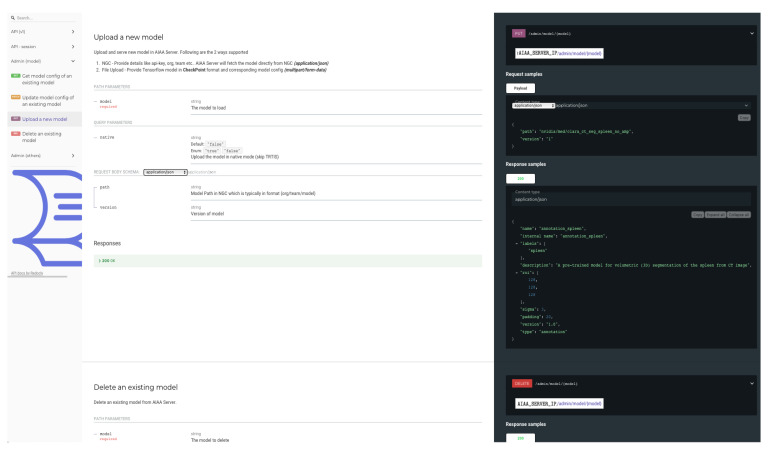
API web page for uploading new models.

**Figure 11:**
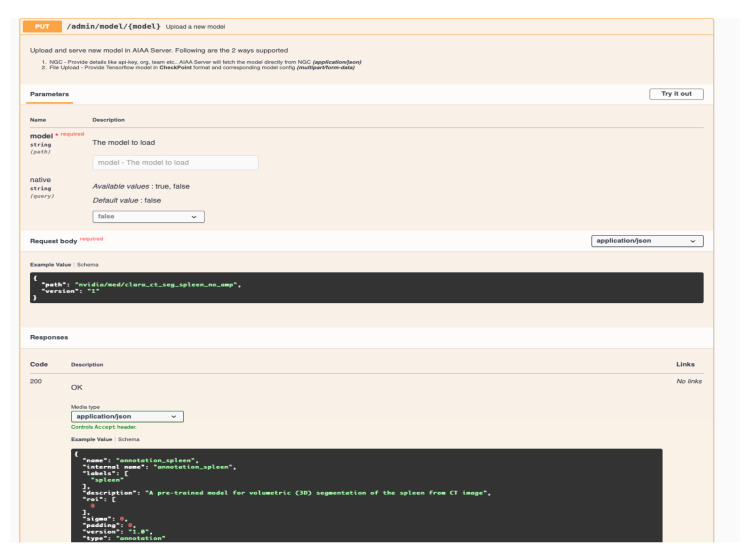
API command for new model uploading to the AIAA server.

In Section 2.3, we detailed the process followed to upload the custom model for ovarian cancer using a *curl* command. It is also possible to upload new models, prepared in the same way, through the AIAA API shown in Figure 10 and Figure 11, replacing the *curl* command with the appropriate API command.

### 3.3. Example: Ovarian Cancer Segmentation

**Figure 12:**
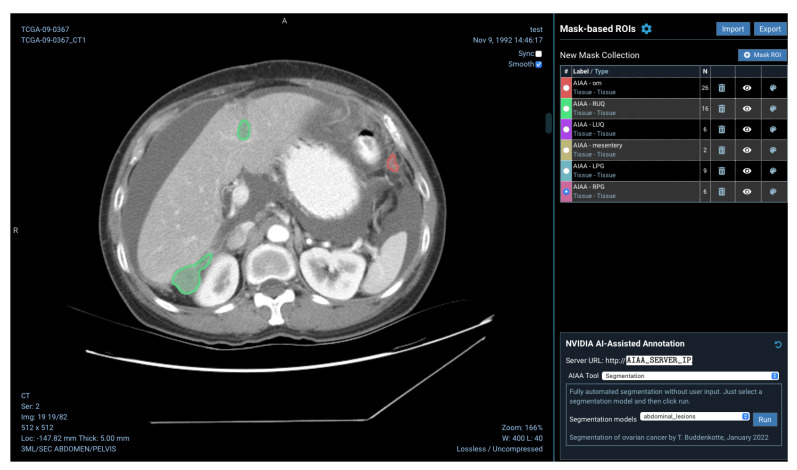
Example 2D CT image with overlaid masks showing the outcome of the custom inference for abdominal lesions added to the pipeline: omentum (red), right upper quadrant (RUQ, green), left upper quadrant (LUQ, purple), mesentery (brown), left paracolic gutter (LPG, blue) and right paracolic gutter (RPG, pink).

For this example, we created AI models for the automated segmentation of ovarian cancer lesions, and incorporated them into our pipeline. An example of the visualisation of one of the three different ovarian cancer models created (detailed in Section 2.3), the one for abdominal lesions, can be found in Figure 12.

### 3.4. Integration of the Radiomics-Based Prediction

The AI inference integrated and run in this pipeline can be for many specific processes. In addition, several processes can be run in chain. In this example, once a segmentation has been automatically obtained with our DL-based model, further measurements can be extracted from the images, and a second inference, this time of a radiomics-based ML model, can be run using the output of the segmentation as input.

**Figure 13:**
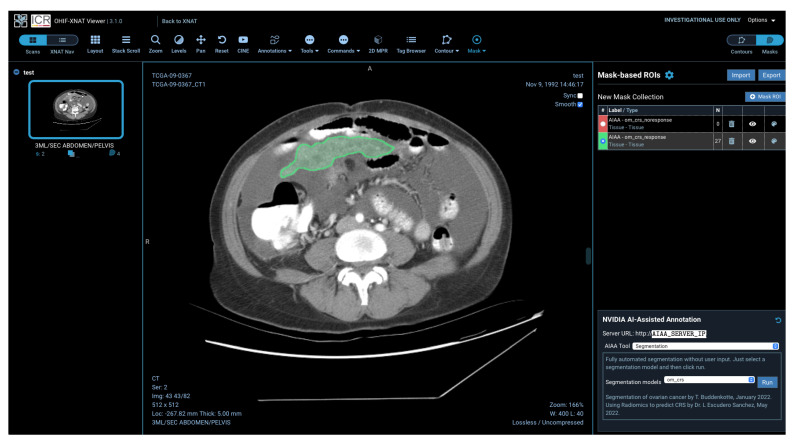
Example outcome of the segmentation and radiomics-based prediction of omental lesions of ovarian cancer, showing the lesions in green as the prediction for this particular patient was to respond to the chemotherapy treatment.

The purpose of this ML-based model is to predict treatment response from the omental lesions, so a special model is created using the pelvis/ovaries + omentum model, discarding the pelvic/ovarian lesions. In addition, this model labelled the output omental lesions as response or non-response, with different colours. An example of the visualisation of the output of this model in the platform can be found in Figure 13.

A video can be found in the Supplementary Material (Video S1) showing a demo of the whole process, from opening an imaging session and selecting the AI model to running and visualising the outcome. The video also shows the output of each step running seamlessly one after another (segmentation, then feature extraction and outcome prediction) written out to the server logs.

## 4. Discussion

In this paper, we have presented a prototype pipeline to incorporate custom AI tools into the clinical research setting, bridging the gap between AI developments and their usage by clinicians. Although this pipeline has the ability to interface and run any type of inference (reconstruction, segmentation, prediction, etc.), we focused on a segmentation + prediction problem as a use case to illustrate its capabilities. This pipeline allows radiologists as end-users to easily access and interact with the latest AI developments, and it also allows AI developers to test, validate and improve their models by having a setting they can manipulate in direct contact with real-world clinical data.

A platform like the one we envision has the potential to democratise the use of AI, as it can increase diversity in training AI models, facilitating their validation and enabling end-users (clinicians) to learn the steps needed to adapt the models to the clinical needs of their institutions. Based on the idea of bringing an AI model to the patient data, instead of patient data to the model, it opens up the capability for clinicians to build, share, locally adapt and validate AI algorithms, while also ensuring patient data stay protected at the local institution. This includes offering tools that vastly simplify training AI models on data from different centres, which helps increase robustness while reducing bias, resulting in improved models across broader populations. A popular way to achieve this is by using federated learning, a way to train AI models against data located in multiple sites without the need to store all the data in the same place or to share it across centres. Instead, models are trained at the source and weights are shared with a single generalised model without the transfer of any data.

We paid special attention to the tumour segmentation task, as the development of AI tools for automated segmentation of organs and tumours has been a clear focus of the AI research community in the last years [20,21,22,23]. These methods do not only reduce the required time to generate such delineations, crucial to perform downstream analyses, but they also have the potential to improve the quality of the segmentations [24] and their reproducibility. Despite their clinical relevance, however, these AI methods are typically developed in technical research groups with little or no communication with the clinical experts who could eventually use them. This creates at present a disconnection for which pipelines, like the one we present in this paper, are of the utmost importance: only by making such AI segmentation tools and their outcomes accessible to radiologists will they fulfil their ultimate purpose, and only by providing a testing environment to AI developers can we achieve the agile development of the necessary methods.

We have also demonstrated that such models for automated segmentation can be easily complemented with other AI tools, such as ML-based radiomic models, to provide clinicians with predictions that can be useful in the clinical setting. For example, we tested incorporating into the pipeline predictions of patient response to chemotherapy treatments, which in the future could be informative to make personalised treatment decisions; such a model could aid in the early identification of patients unlikely to respond to first-line chemotherapy, thereby identifying them as possible candidates for trials of alternative neoadjuvant approaches. With the platform described, the possibilities are manifold to introduce the latest research advances into practical clinical applications in terms of diagnosis, treatment selection and monitoring, in an accessible way for clinicians that will be crucial for their future usage.

In addition, segmentation models widely available today are generally limited to cancer types for which large and often public datasets are available (e.g., brain [25], liver [26], kidney [27]), which results in available models being of the more common cancers only. For other, more rare cancer types, solutions are far from being developed and/or commercialised, as access to images and high-quality annotations to train with is very limited. It is within the research groups in contact with the professionals treating those patients that this can be developed further, and platforms like the one proposed would help to achieve this.

Replicating this pipeline in the clinical context will imply handling biases and limitations from the clinical side, and in particular to overcome the following challenges:

- To ensure the safe usage of potentially identifiable data;
- To acquire and maintain the adequate computing resources required;
- To maintain access for AI developers to the platform for continuous development;
- To seamlessly integrate with existing systems like PACS (picture archiving and communication system) and electronic patient records (EPR) for non-imaging metadata;
- To provide mechanisms for the appropriate presentation of data to clinicians;
- To train clinical staff in the usage of the available tools and to make results of the models available to them in a timely manner;
- To gain the acceptance of patients and to solve any related ethical issues.

In particular, it is necessary to ensure resilience of these AI-based tools against variations in acquisition and instrumentation in order for them to be part of clinical decision support systems for improving diagnostic, prognostic and predictive accuracy. The https://www.fda.gov/medical-devices/software-medical-device-samd/artificial-intelligence-and-machine-learning-aiml-enabled-medical-devices (accessed on 23 June 2023) list of FDA approved DNN-based algorithms for medical image analysis is growing; see also the listing in the https://ericwu09.github.io/medical-ai-evaluation/ (accessed on 23 June 2023) Medical AI Evaluation Database at Nature. However, there are ongoing concerns about the stability of such algorithms, since it is well known that the performance of DNN-based models tends to degrade when the trained model is applied to images with features that deviate somewhat from those used for training. A mathematical motivation for this poor generalisation (lack of stability) is given in [28], and those arguments apply to general DNN-based models. Issues related to poor generalisation arise especially in image analysis tasks that involve elements of visual classification, like semantic segmentation, which are known to be sensitive to variations in texture and contrast [29,30]. Likewise, some radiomic features have been shown to be highly sensitive to variations in several factors, including variability in scanner manufacturer, generation and models, acquisition protocols and reconstruction settings [31,32,33,34]. This lack of robustness that DNN-based image analysis methods and radiomics have against such variations is one reason for the slow dissemination to clinical practice [35,36].

A strategy to improve their generalisation is by data augmentation. This requires a training dataset that is large enough to account for all the variability one expects to encounter. Gathering such large datasets is not only expensive, but it also poses challenges of information governance. Another strategy is to have image analysis methods that encode knowledge about how sensor data are generated, which is now possible thanks to recent advances in physics-informed AI models for image reconstruction [37,38,39,40]. As outlined in [38,41,42], there is a general framework for integrating some of these DNN-based reconstruction methods with DNN-based methods for image analysis. This results in end-to-end approaches for image analysis [43,44,45] that are parametrised by a (handcrafted) model for the underlying physics and a specification of how sensor data are acquired, thus being more resilient against variations in acquisition and instrumentation.

Although much still needs to be carried out to overcome such challenges, we have already provided some solutions with the pipeline presented here. Indeed, we paid attention to build a system that can seamlessly incorporate AI models into intuitive tools for radiologists as end-users, while maintaining the capabilities needed by AI developers for an agile cycle of testing and validation of models.

In the future, we will consider making available to radiologists segmentations together with their uncertainties [46], encouraging them to look more closely at segmented regions where there is lower confidence. We will also expand it to become an end-to-end pipeline optimising reconstruction as well as performing automated tumour segmentation and radiomic-based predictions, applied to other cancers as well in the future. And we will continue working together with clinicians to understand the steps required to bring this pipeline from the research setting to the clinical setting.

## Data Availability

The various elements for this framework are publicly available and can be found in the links provided throughout the text.

## Supplementary Materials

The following supporting information can be downloaded at: https://www.mdpi.com/article/10.3390/diagnostics13172813/s1, Video S1: Demo video (.mov) demonstrating the pipeline in use for the ovarian cancer example, showing the whole process: opening an imaging session, selecting the AI model, running and visualising the outcome, modifying the predicted masks and saving them back to the database.
